# Efgartigimod for the treatment of immune checkpoint inhibitor-associated myocarditis complicated with impending crisis state of myasthenia gravis: a case report

**DOI:** 10.3389/fimmu.2025.1671964

**Published:** 2025-11-28

**Authors:** Mingjia Lu, Ayikemaier Muhetaer, Chenxi Su, Meng Dong, Kariman Aili, Jing Sha, Kaidierya Aimudula, Kailibinuer Abudureheman, Maynur Mamat, Hongyan Li

**Affiliations:** 1 Neuroimmunology Department, Neurology Diagnosis and Treatment Center, People’s Hospital of Xinjiang Uygur Autonomous Region, Urumqi, China; 2 Xinjiang Clinical Research Center for Stroke and Neurological Rare Disease, Urumqi, China; 3 Kashi Prefecture Second People’s Hospital, Kashi, China; 4 Xinjiang Medical University, Urumqi, China

**Keywords:** immune checkpoint inhibitors, immune-related adverse events, myocarditis, impending crisis of myasthenia gravis, myositis

## Abstract

Immune checkpoint inhibitor (ICI)-associated myocarditis is a rare yet life-threatening immune-related adverse event (irAE), whose management is even more challenging when complicated by an impending crisis of myasthenia gravis (MG). This article presents a case of a 69-year-old woman with bladder malignancy, coexisting MG, diabetes, hypertension, and coronary artery disease. On day 16 after tislelizumab (PD-1 inhibitor) therapy, she exhibited aggravated MG symptoms, with an impending crisis, alongside asymptomatic ICI-associated myocarditis. Clinical manifestations included generalized fatigue, anorexia, bilateral ptosis, dysarthria, neck and limb weakness, shortness of breath, and dyspnea. Laboratory tests revealed elevated troponin T, troponin I, creatine kinase (CK), creatine kinase–myocardial band (CK-MB), aspartate aminotransferase (AST), and alanine aminotransferase (ALT) levels, suggesting a diagnosis of ICI-associated myocarditis and liver injury. Doppler ultrasound indicated thrombosis in the left calf muscle space. Treatment with efgartigimod (10 mg/kg) significantly improved myasthenic symptoms, with Myasthenia Gravis–Activities of Daily Living (MG-ADL) and Quantitative Myasthenia Gravis (QMG) scores decreasing from 11 to 5 and 26 to 11, respectively. Treatment with prednisone acetate (60 mg once daily) resulted in gradual reductions in myocardial enzymes, troponins, and transaminases. Efgartigimod demonstrated notable efficacy as salvage therapy for ICI-associated myocarditis, myositis, liver injury, and impending crisis of MG. Early recognition and tailored treatment strategies are crucial for improving patient outcomes in such complex cases.

## 1 Introduction

As a major breakthrough in cancer immunotherapy, immune checkpoint inhibitors (ICIs) restore T cell-mediated antitumor immune activity by blocking inhibitory signaling pathways such as programmed death-1 (PD-1), programmed death-ligand 1 (PD-L1), and cytotoxic T-lymphocyte antigen-4 (CTLA-4), thereby significantly improving the survival rates of patients with various malignancies (1–3). With the widespread application of ICIs in diseases such as melanoma, non-small cell lung cancer, renal cell carcinoma, gastric cancer, and lymphoma, their clinical efficacy has been fully demonstrated (4–7).

However, the overactivation of the immune system also leads to multi-organ immune-related adverse events (irAEs), which can affect almost all major systems (8, 9). Common irAEs include rash, colitis, hepatitis, endocrine abnormalities, and pneumonitis (10). Among these, adverse reactions involving the cardiovascular and neuro-muscular systems, although relatively rare, are often associated with severe clinical consequences and can even be life-threatening (7, 11).

Among the numerous severe irAEs, immune checkpoint inhibitor-associated myocarditis, though rare, carries a high fatality rate. According to multiple studies, its overall incidence is approximately 0.1%–1%, with a clinical mortality rate ranging from 25% to 50% (12–14) Most cases occur within 2–6 weeks after ICI treatment and often present with symptoms such as chest pain, fatigue, dyspnea, arrhythmias, and even sudden cardiac death (15, 16). The pathological mechanism primarily involves T cell-mediated myocardial injury and the disruption of immune tolerance (17, 18). Different types of ICIs vary in their risk of inducing myocarditis: PD-1 or PD-L1 inhibitors alone can cause myocarditis, but the risk is significantly higher with combination therapy involving PD-1/PD-L1 and CTLA-4 inhibitors. For example, the regimen combining Nivolumab and Ipilimumab shows a markedly increased incidence of myocarditis compared to monotherapy, suggesting that multi-pathway immune activation may lead to synergistic toxic effects (19, 20).

It is noteworthy that ICI-associated myocarditis is often accompanied by myositis or myasthenia gravis (MG), forming what is referred to as an “immune-related neuro-muscular overlap syndrome.” Literature reports indicate that approximately 30%–40% of ICI-associated myocarditis cases are complicated by myositis, and about 10% are accompanied by MG (21, 22). Patients with such “triad syndromes” (MG–myositis–myocarditis) often experience rapid disease progression, with a mortality rate exceeding 50% (21). Previous cases have predominantly been observed in patients treated with PD-1 or PD-L1 inhibitors (such as Nivolumab and Pembrolizumab), clinically manifesting as progressive muscle weakness, respiratory failure, and cardiac dysfunction (23, 24).

Although there have been a limited number of reported cases of ICI-induced myasthenia gravis (MG) accompanied by myocarditis, the co-occurrence of myasthenic crisis remains extremely rare, and there is still no consistent consensus regarding immunomodulatory treatment strategies for this condition. Efgartigimod, as a novel FcRn antagonist, accelerates the degradation of pathogenic IgG antibodies and has demonstrated favorable safety and efficacy in the treatment of myasthenia gravis. However, no systematic studies have yet reported on the use of Efgartigimod for treating ICI-associated MG with myocarditis.

This study reports a case of asymptomatic myocarditis accompanied by liver function impairment and myasthenic crisis following treatment with immune checkpoint inhibitors. The patient showed significant improvement after the combined use of Efgartigimod in addition to conventional immunosuppressive therapy. This case not only further enriches the clinical spectrum of ICI-induced immune-related myocarditis and MG overlap syndrome but also provides new insights and clinical references for the management of such complex immune toxicities.

## 2 Methods

### 2.1 Case presentation and methods

This case involves a 69-year-old female patient admitted with complaints of right eyelid ptosis for 2 years, neck weakness for 1 week, and slurred speech for 2 days. The patient was previously diagnosed with bladder cancer (CT2NxMx) in 2018 and underwent transurethral resection of bladder tumor along with intravesical gemcitabine perfusion therapy. After recurrence in 2024, she received three cycles of gemcitabine combined with cisplatin chemotherapy. On January 9, 2025, she was treated with the programmed death-1 (PD-1) inhibitor tislelizumab (200 mg, intravenous injection).

### 2.2 Clinical data collection

Patient clinical data were obtained from the electronic medical record system, including past medical history, oncology treatment history, ICI medication information, and symptom progression. The severity of myasthenia gravis (MG) was assessed using the Myasthenia Gravis Foundation of America (MGFA) classification criteria and quantified through MG-ADL and QMG scores.

### 2.3 Laboratory and immunological tests

Laboratory tests included complete blood count, liver and kidney function, myocardial enzyme profile (CK, CK-MB, LDH), myoglobin, troponin I/T, and N-terminal pro-B-type natriuretic peptide (NT-proBNP). Immunological testing employed enzyme-linked immunosorbent assay (ELISA) to detect acetylcholine receptor antibodies (AChR-Ab), with anti-MuSK antibody testing performed if clinically indicated. Results showed positivity for AChR-Ab (4.687 nmol/L,normal values<0.5nmol/L).

### 2.4 Imaging and cardiac evaluation

The patient underwent 12-lead electrocardiography and echocardiography during the period of worsened myasthenia, revealing mild cardiac dysfunction. Further cardiac magnetic resonance (CMR) imaging indicated mild myocardial edema without significant late gadolinium enhancement (LGE). In combination with elevated myocardial enzymes and after ruling out acute coronary syndrome and infection, the patient was diagnosed with immune checkpoint inhibitor-associated myocarditis (ICI-associated myocarditis), characterized as non-fulminant and mild. The diagnosis was made in accordance with the European Society of Cardiology (ESC, 2021) myocarditis guidelines. Additionally, pulmonary function tests showed a decrease in forced vital capacity (FVC) to 64.7%, and venous ultrasound of the lower limbs indicated thrombosis in the muscular venous plexus of the left calf.

### 2.5 Treatment course

Upon admission, the patient presented with impending crisis of myasthenia gravis (MG), characterized by shallow and rapid breathing and significant neck muscle weakness. On the day of admission, Efgartigimod was administered according to a weight-based dosing regimen (10 mg/kg, approximately 600 mg, intravenous infusion), once weekly for a total of four cycles, following the standards of the ADAPT clinical trial (Howard et al., Lancet Neurol 2021). Muscle strength, respiratory function, and myocardial enzyme levels were monitored before and after each administration. The next day, significant improvement in dyspnea and neck muscle strength was observed. As the patient’s family declined high-dose glucocorticoid pulse therapy, oral prednisone acetate (60 mg/day) was initiated instead, based on the MGFA-recommended initial dose (1 mg/kg/day). After symptom relief, a standardized tapering protocol was started at week 4, reducing the dose by 5–10 mg every two weeks. When the dose reached 40 mg/day, the tapering interval was extended to monthly reductions. Throughout the tapering process, MG-ADL scores, myocardial enzyme levels, and liver function were closely monitored. During treatment, myasthenic symptoms gradually improved: the MG-ADL score decreased from 11 to 4, and the QMG score dropped from 26 to 8. Myocardial enzyme and troponin levels significantly declined, and liver function returned to normal. The treatment plan was developed collaboratively by a multidisciplinary team (MDT) in accordance with the MGFA and ASCO guidelines for the management of immune-related adverse events.

### 2.6 Follow-up and prognosis

The patient showed significant symptomatic improvement after in-hospital treatment, with no recurrence of respiratory failure or arrhythmia. Monthly follow-ups were conducted after discharge, with reassessment of myocardial enzyme profiles, AChR antibody titers, and liver function. After three months, the patient’s condition remained stable with no signs of recurrence.

## 3 Case report

A 69-year-old female patient was admitted with right eyelid ptosis for 2 years, weakness during neck extension for 1 week, and dysarthria for 2 days as the chief complaints. In February 2023, the patient developed right eyelid ptosis (3:00-9:00 direction), characterized by mild symptoms in the morning and worsening with fatigue. During an outpatient visit at our hospital, a fatigue test was positive, and anti–acetylcholine receptor antibody (anti-AChR) level was 4.687 nmol/L. The patient was not compliant with prescribed pyridostigmine therapy. In 2018, the patient was diagnosed with bladder cancer (CT2NxMx) and underwent transurethral resection of the bladder tumor, followed by intravesical gemcitabine therapy, resulting in stable disease. In 2024, cystoscopy revealed tumor recurrence, and three cycles of gemcitabine plus cisplatin chemotherapy were administered to the patient, starting in November 2024. On January 9, 2025, the patient underwent treatment with tislelizumab at 200 mg intravenously. On January 25, 2025, the patient developed right eyelid ptosis, which resolved spontaneously after one week but was followed by complete left eyelid ptosis, diplopia, and neck weakness.On February 8, 2025, the symptoms worsened, including dysarthria, generalized fatigue, chest tightness, shortness of breath, orthopnea, and weak cough reflex, prompting admission to our hospital. past medical history was: hypertension and primary aldosteronism for 20 years, type 2 diabetes mellitus for 8 years. Notably, the patient had no statin treatment within the preceding three months.

**Figure 1:**
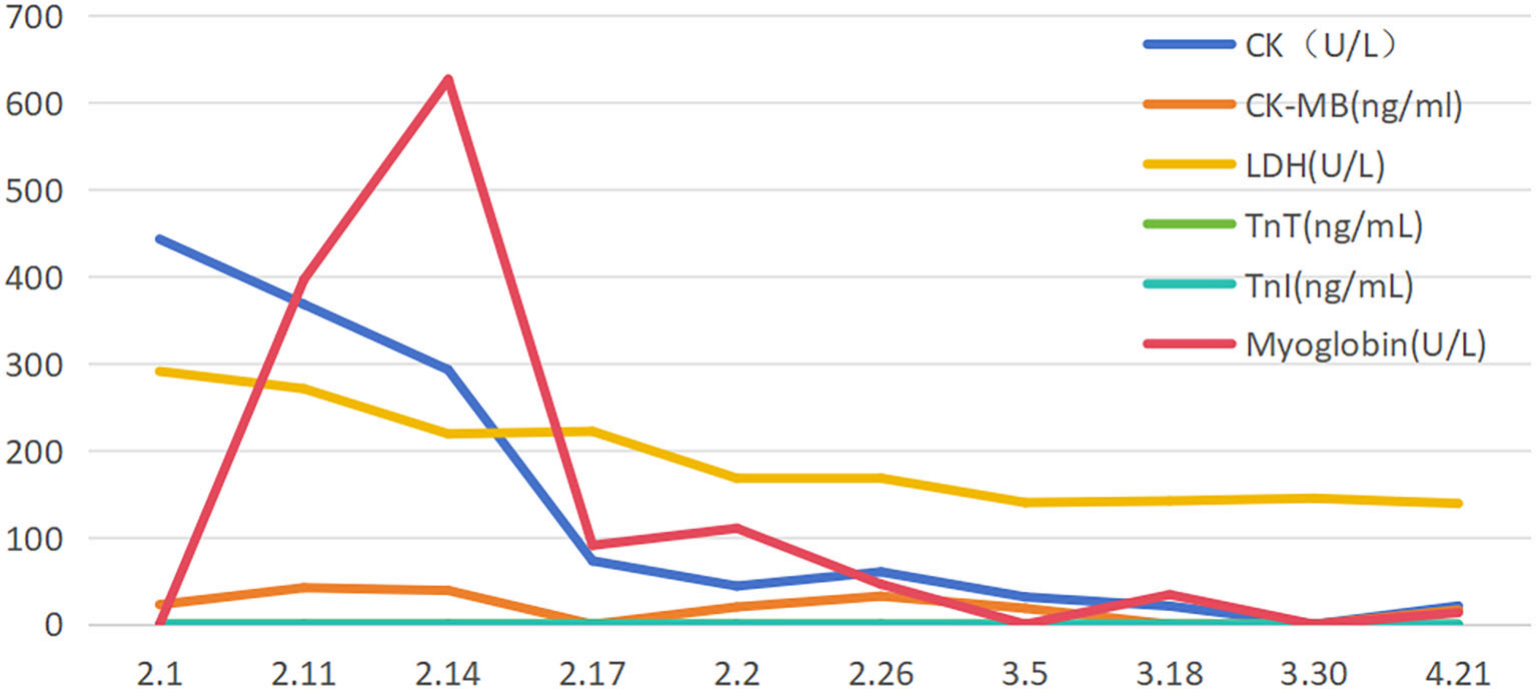
Changes in cardiac injury markers, myoglobin, and liver function.

**Figure 2:**
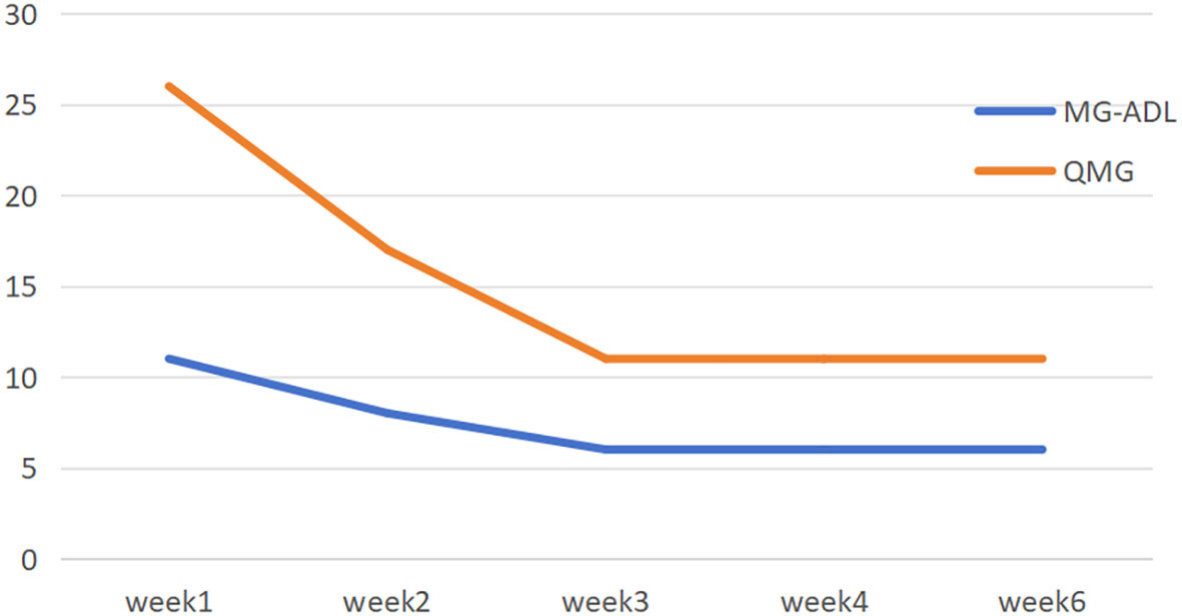
Trend changes in MG-ADL and QMG scores.

**Figure 3:**
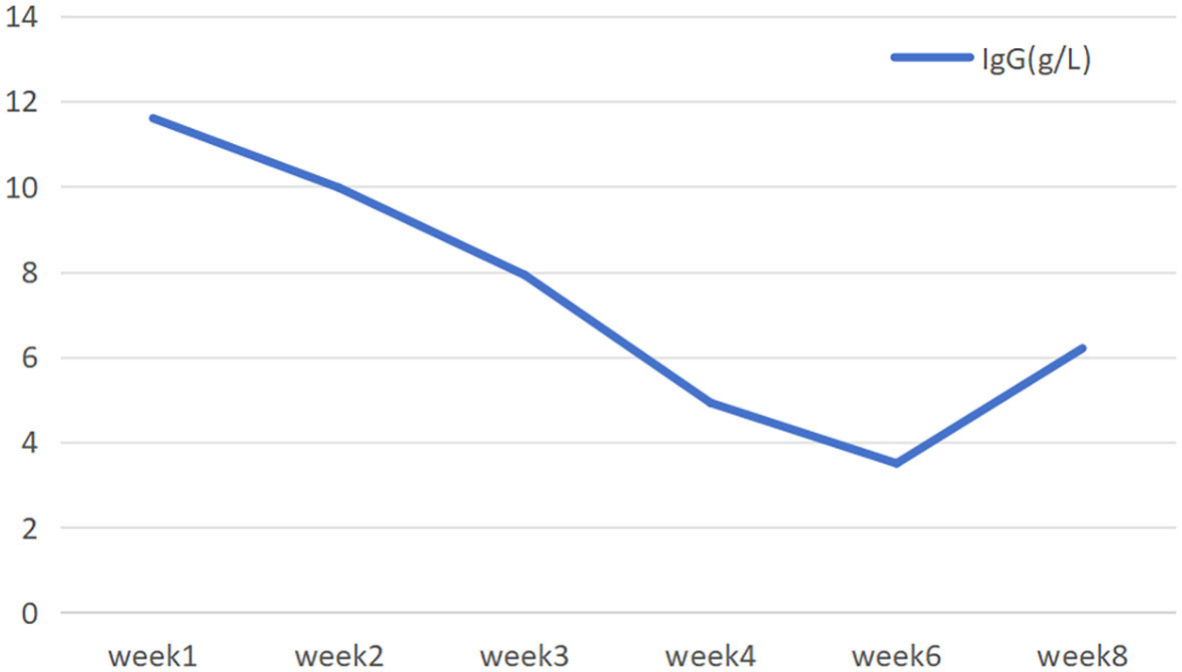
Trend changes in IgG.

Neurological examination revealed complete left eyelid ptosis, right eyelid ptosis (1:00-11:00 direction), elevated position of the left eye with restricted upward and downward gaze, dysarthria, weakened pharyngeal reflex, neck muscle strength of grade III, limb muscle strength of grade IV,. MG-ADL and QMG scores were 11 and 26 at admission, respectively.Arterial blood gas analysis showed pH at 7.447, pCO_2_ at 35.1 mmHg, pO_2_ at 70.8 mmHg, and SpO_2_ at 92.9%.Liver function tests revealed elevated alanine aminotransferase (51 U/L,normal values 0-35U/L) and aspartate aminotransferase (36 U/L,normal values 14-36U/L) levels. Cardiac biomarkers were notably elevated, including creatine kinase (443 U/L,normal values 30-150U/L), lactate dehydrogenase (291 U/L,normal values 120-246U/L), CK-MB (22.7 ng/mL,normal values ≤ 5ng/ml), myoglobin (618 μg/L,normal values 0-106U/L), troponin I (0.051 ng/mL,normal values 0-0.026ng/mL), and troponin T (0.355 ng/mL,normal values 0-0.014ng/mL). Lower extremity venous ultrasound identified thrombosis in the left calf muscular venous plexus. Pulmonary function tests demonstrated FVC 64.7%. The patient was diagnosed with impending crisis State of myasthenia gravis on admission day and immediately administered intravenous efgartigimod at 0.6g (10 mg/kg), completing a total of 4 cycles. Concurrently, prednisone 60 mg/day was administered, with tapering initiated after 4 weeks at a rate of 5 mg reduction every two weeks. By the 6th week, the prednisone dosage was reduced to 40 mg/day, with sustained symptomatic improvement. By the following day, dyspnea and neck muscle strength were improved. The newly elevated troponins (T and I) and cardiac enzymes, after excluding acute infection and acute coronary syndrome, confirmed the diagnosis of immune checkpoint inhibitor-associated myocarditis, classified as non-fulminant and mild in severity with concomitant liver injury. Given the complexity of the patient’s condition and multiple comorbidities, a hospital-wide multidisciplinary consultation was organized. Following thorough discussion and review of relevant reports (4, 5), the team established diagnostic criteria and treatment protocols for ICI-associated myocarditis and recommended high-dose steroid pulse therapy. However, this treatment approach was declined by her family, and oral prednisone acetate (60 mg) once daily was initiated instead. During the treatment, myasthenic symptoms in the patient improved significantly, with corresponding decreases in cardiac enzymes and troponins and normalization of liver function indexes. Serial follow-up assessments revealed a favorable and sustained therapeutic response (**Figures 1**–**3**). A line graph illustrates the temporal trends of six biomarkers: myoglobin surged to 600 on day 2.14 before declining sharply; creatine kinase (CK) demonstrated a steady decrease; lactate dehydrogenase (LDH) fluctuated around 300 U/L; troponin I (TnI) peaked at 100 U/L; while both CK-MB and troponin T (TnT) remained consistently below 100 U/L throughout the observation period. Cardiac injury markers, including CK, CK-MB, LDH, TnI, TnT, and myoglobin, were markedly elevated during the acute phase and peaked around February 14. After initiation of immunotherapy and supportive treatment, these markers rapidly declined and returned to normal levels by late February, indicating effective resolution of myocardial involvement. Neuromuscular function improved in parallel, as reflected by a steady reduction in MG-ADL and QMG scores from week 1 (11 and 26, respectively) to week 3 (6 and 11), with both scores remaining stable thereafter through week 6. This pattern demonstrates significant and sustained recovery of muscle strength and functional capacity. Serum IgG levels showed a gradual decline from 11.6 g/L(normal values 7.51-15.60g/L) at week 1 to 3.5 g/L by week 6, followed by a mild rebound to 6.2 g/L at week 8, consistent with the expected pharmacodynamic profile of immunotherapy and subsequent stabilization of humoral immunity. Overall, these longitudinal observations highlight continuous biochemical, functional, and immunologic improvement throughout the treatment and follow-up periods.

## 4 Discussion

Tislelizumab, a representative PD-1 inhibitor independently developed in China with global approval, is extensively prescribed for Chinese cancer patients. Immune checkpoint inhibitors work by releasing T-cell inhibitory signals and enhancing immune responses, thereby inducing tumor cell elimination. However, they may also trigger the immune system to attack self-tissues, leading to immune-related adverse events (irAEs). This report describes a patient who developed recurrent and gradually worsening MG symptoms 16 days after PD-1 inhibitor administration, progressing to impending crisis status upon hospitalization. Evaluation at admission revealed asymptomatic elevations in cardiac enzymes (troponins T and I), hepatic dysfunction, and concomitant lower extremity venous thrombosis. Given the critical impending crisis of MG status in the current case, immediate salvage therapy with efgartigimod (0.6g) was initiated for antibody clearance, resulting in symptomatic improvement. Due to disease complexity and severity, a multidisciplinary team was convened. According to the European Society of Cardiology (ESC, 2021) myocarditis diagnostic guidelines and the criteria for ICI-associated myocarditis proposed by Bonaca et al. (18, 25, 26), the diagnosis must meet at least one of the following conditions: (1) elevated biomarkers of myocardial injury (e.g., cTnI or cTnT); (2) imaging evidence (cardiac MRI showing edema or LGE); (3) exclusion of other explainable causes; and (4) a history of ICI exposure. This patient met multiple criteria: significant elevation of troponin T and I, cardiac MRI showing mild edema, and exclusion of other causes such as acute coronary syndrome, viral infection, and drug-induced myocardial injury, strongly supporting the diagnosis of ICI-associated myocarditis. Consensus diagnosis confirmed PD-1 inhibitor-induced myocarditis with MG exacerbation, recommending high-dose corticosteroid pulse therapy. After thorough deliberation with the patient’s family who declined pulse therapy, oral prednisone acetate (60 mg daily) was administered, combined with weekly efgartigimod (0.6g IV). At one-month follow-up, stable disease was achieved, with prednisone tapered to 40 mg daily and progressive normalization of cardiac injury markers. In this case, the dosage and tapering strategy of prednisone followed the MGFA guideline-recommended immunosuppressive treatment regimen, starting with an initial dose of approximately 1 mg/kg/day and subsequently tapering at a standard rate of 5–10 mg reduction every two weeks to minimize the risk of relapse and maintain efficacy. Efgartigimod was administered intravenously at a weight-based dose of 10 mg/kg once weekly for a total of four cycles. This regimen was based on the ADAPT study, which validated its safety and rapid onset of action in AChR antibody-positive myasthenia gravis patients. The combination therapy in this case achieved rapid relief of MG symptoms while avoiding the side effects associated with high-dose steroid pulse therapy, demonstrating good clinical reproducibility.

The pathological basis of the overlap between ICI-associated myocarditis and MG primarily involves cross-reactive antigen responses and T-cell dysregulation. The PD-1 pathway plays a critical role in maintaining peripheral immune tolerance, and its blockade can lead to T-cell recognition of shared antigens in skeletal and cardiac muscle, triggering cytotoxic T lymphocyte-mediated tissue damage. Furthermore, studies have shown elevated levels of autoantibodies (such as AChR-Ab and anti-striated muscle antibodies) in patients with ICI-associated MG and myocarditis, indicating that abnormal humoral immunity also contributes to the pathogenesis. The expression of similar antigens in cardiac and skeletal muscle, such as myosin and troponin, may explain the clinical overlap between MG and myocarditis (27–29).

Efgartigimod is a selective, novel FcRn (neonatal Fc receptor) antagonist. FcRn plays a critical role in the recycling and prolongation of the half-life of IgG antibodies. By blocking the binding of IgG to FcRn, Efgartigimod promotes the degradation of pathogenic IgG antibodies (such as anti-AChR antibodies and anti-striated muscle antibodies) in the circulation, thereby rapidly reducing antibody levels and mitigating antibody-mediated tissue damage. Compared to conventional plasma exchange (PLEX) or intravenous immunoglobulin (IVIg), Efgartigimod offers advantages such as convenient intravenous administration, rapid onset of action, and a lower risk of immunosuppression, making it particularly suitable for patients with thrombosis or contraindications to IVIg (30, 31). Notably, a recent study reported the use of Efgartigimod in a patient with stiff-person syndrome (SPS) comorbid with myasthenia gravis, which demonstrated significant improvement in both SPS and MG symptoms over a 12-week treatment cycle (32). This study is the first to confirm the therapeutic potential of FcRn antagonists in antibody-mediated neurological diseases beyond MG, further supporting their mechanism of precise immune modulation through selective clearance of pathogenic IgG antibodies. The application of Efgartigimod in this case also yielded remarkable efficacy, suggesting that FcRn blockade is not only effective in managing MG crises but may also help alleviate antibody-mediated immune responses in ICI-associated myocarditis, offering a new therapeutic direction for such complex immune toxicities.

To date, a limited number of literature reports have documented cases of ICI-induced MG with concomitant myocarditis. A systematic review of 60 IM3OS cases revealed that 60% of the patients died during hospitalization. The majority of patients developed symptoms after just a single dose of ICI therapy, indicating the aggressive nature of such adverse reactions and underscoring the need for early recognition and treatment (21). A study indicates that ICI-associated MG progresses more rapidly and severely, often accompanied by myocarditis and myositis, highlighting the critical need for early recognition and intervention during its “window period” (33). Another case report demonstrated that ICI-associated myocarditis may initially present as cardiac conduction block, subsequently complicated by MG and myositis, leading to rapid clinical deterioration and fatal outcome (34).

To rule out other potential causes, the patient underwent a comprehensive evaluation after admission. At the time of elevated cardiac enzymes, the patient was not on statins or cardiotoxic medications. Virological screening (including tests for Coxsackie virus, EBV, CMV, influenza A, etc.) returned negative results, and no imaging features indicative of toxic or ischemic myocardial injury were detected. Considering the history of ICI exposure and the clinical progression, the patient was ultimately diagnosed with PD-1 inhibitor-induced immune-related myocarditis.

Previous evidence indicates that MG patients with concurrent myocarditis have a mortality rate as high as 50%, irrespective of ICI exposure (6).Regarding therapeutic management, immunosuppression remains the cornerstone treatment option for ICI-associated myocarditis, myositis, and MG exacerbations, with corticosteroids serving as the first-line drugs per international guidelines (5). In the present case, efgartigimod was employed for salvage therapy to alleviate myasthenic symptoms, while prednisone acetate was administered for immunomodulation, as a strategy circumventing the adverse effects of high-dose steroid pulses while preventing further MG deterioration. While traditional salvage therapeutic options for impending crisis of MG include plasma exchange (PLEX) and intravenous immunoglobulin (IVIG), the latter was contraindicated in this patient due to left calf muscular venous thrombosis. PLEX carries inherent risks such as central venous access requirement, plasma product exposure, and anaphylactic potential. In contrast, efgartigimod provides practical advantages with convenient intravenous administration, rapid onset, and effective pre-crisis intervention, potentially averting intubation and intensive care admission.

With the expanding population of cancer patients treated with increasingly prevalent immunotherapy, particular vigilance is warranted when administering ICIs to MG patients with malignancies. Clinicians must maintain a high level of awareness for irAEs, implement early detection protocols, and engage multidisciplinary teams for prompt intervention, which are all critical factors for optimizing patient outcomes. This report underscores the importance of coordinated, preemptive management in this high-risk patient population.

## Data Availability

The original contributions presented in the study are included in the article/**Supplementary Material**. Further inquiries can be directed to the corresponding author.
