# Supplementary material for: Efgartigimod for the treatment of immune checkpoint inhibitor-associated myocarditis complicated with impending crisis state of myasthenia gravis: a case report
Source: Front Immunol. 2025 Nov 28;16:1671964. doi: 10.3389/fimmu.2025.1671964 (PMC12698593; doi:10.3389/fimmu.2025.1671964)
Supplement: Supplementary file 1 [file Table1.docx]

### ****1. Participant Selection Criteria****

#### ****Inclusion Criteria****

Clearly define the target population.

Eligible participants were adults (≥18 years) with immune checkpoint inhibitor-associated myocarditis (ICI-myocarditis) and pre-crisis myasthenia gravis (MG), confirmed by diagnostic criteria, elevated troponin, positive anti-AChR antibodies

#### ****Exclusion Criteria****

"Patients with end-stage organ failure, concurrent infections, or prior use of efgartigimod were excluded."

### ****2. Recruitment Methods****

"The patient was referred to our tertiary care center due to refractory ICI-myocarditis complicated by MG. Informed consent was obtained for off-label efgartigimod use and publication."

### ****3. Justification for Study Population****

"ICI-myocarditis with MG is a rare but life-threatening complication. This population was selected to evaluate efgartigimod’s dual immunomodulatory effects on both conditions, as no standard therapy exists."

"Early intervention in pre-crisis MG may prevent respiratory failure, justifying the focus on this high-risk subgroup."

"Our institution is a regional referral center for ICI toxicity, ensuring adequate patient volume for this rare condition."

### ****4. Ethical Considerations****

"To avoid selection bias, all eligible patients during the study period were invited to participate. Non-English speakers were provided translated consent forms."

We enrolled adults with ICI-myocarditis and pre-crisis MG (defined by [criteria]) from the Neurology Department at Xinjiang Uygur Autonomous Region People’s Hospital between January 2023 and December 2023. Participants were identified via EMR alerts for elevated troponin and MG exacerbations. Exclusion criteria included active infections or prior use of complement inhibitors. This population was chosen due to the unmet need for targeted therapies in ICI-myocarditis with concurrent MG.
