# Supplementary material for: Efgartigimod for the treatment of immune checkpoint inhibitor-associated myocarditis complicated with impending crisis state of myasthenia gravis: a case report
Source: Front Immunol. 2025 Nov 28;16:1671964. doi: 10.3389/fimmu.2025.1671964 (PMC12698593; doi:10.3389/fimmu.2025.1671964)
Supplement: Supplementary file 2 [file Table2.docx]

"The study protocol was approved by the Ethics Committee of Xinjiang Uygur Autonomous Region People’s Hospital (Approval No. KY2025041103). Informed consent was obtained from the patient for publication of this case report, including clinical details and images."

"Participants were informed about the experimental nature of efgartigimod, potential cardiac monitoring requirements, and their right to discontinue participation. All data were de-identified to protect privacy."

Consent was obtained during a face-to-face meeting with a researcher, who provided a written information sheet and answered questions. Participants signed the consent form after a 24-hour reflection period."*

"For this database analysis, the Ethics Committee waived individual consent due to the use of anonymized historical data ."

"The patient provided written consent for publication, including use of medical images and identifiable clinical details."

All procedures followed the Declaration of Helsinki and Good Clinical Practice (GCP) guidelines."

"Signed consent forms are retained by the investigating institution and available for review upon request."
