# Supplementary material for: Efgartigimod for the treatment of immune checkpoint inhibitor-associated myocarditis complicated with impending crisis state of myasthenia gravis: a case report
Source: Front Immunol. 2025 Nov 28;16:1671964. doi: 10.3389/fimmu.2025.1671964 (PMC12698593; doi:10.3389/fimmu.2025.1671964)
Supplement: Supplementary file 3 [file Table3.docx]

Informed Consent for Case Report Publication

Title of Case Report:Efgartigimod for the Treatment of Immune Checkpoint Inhibitor-Associated Myocarditis Complicated with Pre-Crisis State of Myasthenia Gravis: A Case Report

1.Purpose of Publication

This case report aims to share unique clinical findings, immunological insights, or therapeutic outcomes to advance medical knowledge.

2.Voluntary Participation

The patient (or legal guardian) voluntarily consents to:

- Publication of de-identified clinical details (labs, imaging, treatment).

- Use of anonymized images (e.g., histopathology, radiology) with no identifiable features.

3.Confidentiality Assurance

- All personal identifiers (name, birthdate, hospital ID) will be removed.

- Data will be stored securely and used solely for academic purposes.

4.Risks:Minimal (potential loss of anonymity despite efforts).

- Benefits:Contributes to scientific progress; may help future patients.

5.The patient may withdraw consent before manuscript submission without affecting medical care.

6.I understand the above information and provide consent for publication of this case report in Frontiers in Immunology and related academic platforms.

I will receive a signed copy of the informed consent.

（Patient's name）： ________________________

（Signature of patient）：_________________________

（Signature of doctor）：_________________________

（Date）：______ ______
