# Supplementary material for: Efgartigimod for the treatment of immune checkpoint inhibitor-associated myocarditis complicated with impending crisis state of myasthenia gravis: a case report
Source: Front Immunol. 2025 Nov 28;16:1671964. doi: 10.3389/fimmu.2025.1671964 (PMC12698593; doi:10.3389/fimmu.2025.1671964)
Supplement: Supplementary file 4 [file DataSheet1.pdf]

Xinjiang Uygur Autonomous Region People's Hospital Clinical Research Ethics Committee Review Opinion

Research Protocol Title	Efgartigimod for the Treatment of Immune Checkpoint Inhibitor-Associated Myocarditis Complicated with Pre-Crisis State of Myasthenia Gravis: A Case Report		
KY2025032101	KY2025041103	Department	Neurology
Principal Investigator	Lu Mingjia	Title	Associate Chief Physician
Project Source			
Review Category	☑ Initial Review □ Follow-up Review □ Re-review □ Progress Report	Review Method	□ Full Committee Review ☑ Expedited Review
Key Review Documents	1. Research Ethics Application Form<br>2. Paper		
Voting Results	Meeting Date: /Year/Month/Day, Total Members: /, Present: /, Among Them: Voters: /, Recusals: /.		
	Among Them: Approved: /, Approved with Modifications: /, Re-review After Modifications: /, Continue Research: /, Disapproved: /, Suspended or Terminated: /.		
	Conclusion: ☑ Approved □ Approved with Modifications □ Re-review After Modifications □ Disapproved □ Suspended or Terminated		
Review Opinion	Is Continuous Review Required: □Yes ☑No	Review Frequency from Approval Date: □ 6 Months □12 Months ☑ Other: No Follow-up Review Required	
Chairperson's Signature	李[illegible]		
Contact Phone	0991-8568013		
Ethics Committee Seal			
Date	11 April 2025		

CS 扫描全能王
3亿人都在用的扫描App
